# Antimicrobial PVA Hydrogels with Tunable Mechanical Properties and Antimicrobial Release Profiles

**DOI:** 10.3390/jfb14040234

**Published:** 2023-04-20

**Authors:** Caitlyn Greene, Henry T. Beaman, Darnelle Stinfort, Maryam Ramezani, Mary Beth B. Monroe

**Affiliations:** Department of Biomedical and Chemical Engineering, BioInspired Syracuse: Institute for Material and Living Systems, Syracuse University, Syracuse, NY 13244, USA

**Keywords:** hydrogel, shape memory polymer, phenolic acid, antimicrobial

## Abstract

Hydrogels are broadly employed in wound healing applications due to their high water content and tissue-mimicking mechanical properties. Healing is hindered by infection in many types of wound, including Crohn’s fistulas, tunneling wounds that form between different portions of the digestive system in Crohn’s disease patients. Owing to the rise of drug-resistant infections, alternate approaches are required to treat wound infections beyond traditional antibiotics. To address this clinical need, we designed a water-responsive shape memory polymer (SMP) hydrogel, with natural antimicrobials in the form of phenolic acids (PAs), for potential use in wound filling and healing. The shape memory properties could allow for implantation in a low-profile shape, followed by expansion and would filling, while the PAs provide localized delivery of antimicrobials. Here, we developed a urethane-crosslinked poly(vinyl alcohol) hydrogel with cinnamic (CA), p-coumaric (PCA), and caffeic (Ca-A) acid chemically or physically incorporated at varied concentrations. We examined the effects of incorporated PAs on antimicrobial, mechanical, and shape memory properties, and on cell viability. Materials with physically incorporated PAs showed improved antibacterial properties with lower biofilm formation on hydrogel surfaces. Both modulus and elongation at break could be increased simultaneously in hydrogels after both forms of PA incorporation. Cellular response in terms of initial viability and growth over time varied based on PA structure and concentration. Shape memory properties were not negatively affected by PA incorporation. These PA-containing hydrogels with antimicrobial properties could provide a new option for wound filling, infection control, and healing. Furthermore, PA content and structure provide novel tools for tuning material properties independently of network chemistry, which could be harnessed in a range of materials systems and biomedical applications.

## 1. Introduction

Crohn’s Disease (CD), a major type of inflammatory bowel disease (IBD), which affects >1,000,000 people in the U.S. and Europe alone [1]. A common side effect of CD is the formation of tunneling wounds between portions of the digestive tract, known as fistulas. When diagnosed with CD, ~20% of patients already have a fistula, and ~35% of CD patients will eventually develop fistulas in their lifetime [2]. Beyond the use of immunosuppressants, current treatment options include setons and fistula fillers. These treatments are limited by invasive surgeries, dislodging, poor fecal incontinence, and infection [3,4,5,6]. Although current treatments can be effective in the short-term, post-operative recurrence rates are 33% at 5 years, and many patients ultimately require invasive bowel restrictive surgery [7]. 

Another non-invasive approach to fistula treatment involves the use of antibiotics. Colonization of the gut by harmful bacteria, such as invasive *Escherichia coli* (*E. coli*), has been linked to IBD [8]. The most commonly employed antibiotic in CD is metronidazole, which treats overgrown anaerobic bacteria in the fistula tract [9]. Ciprofloxacin, another antibiotic, is common but has a wider range of applications and fewer side effects [10]. Continued antibiotic treatment improves fistula healing over extended periods of time, indicating that infection plays a role in fistula persistence, but upon completion of the treatment, the fistula will often reform [11]. Continued treatment with antibiotics is not advised due to the potential for the development of drug-resistant bacteria. The use of natural antimicrobials could solve this issue, as they are less likely to promote resistance, but these natural chemicals are often not as effective at infection treatment when systemically administered [10]. For improved efficiency of natural antimicrobials, localized sustained delivery, such as that seen with other CD treatments (e.g., infliximab), would be ideal [12].

One potential source of natural antimicrobials is phenolic acids (PAs). PAs show a wide range of antimicrobial effects on multiple bacteria, including drug-resistant strains [13,14,15]. These secondary plant-based metabolites can either kill or inhibit the growth of bacteria. Common PAs are based on benzoic or cinnamic acid, in which the benzene ring is substituted with hydroxyls. Other side groups, such as alkyl ethers, are also common on these natural antimicrobials. Studies show that PA side groups can affect antibacterial, reactive oxygen species scavenging, and cytotoxic properties [16]. Other properties of PAs that may be beneficial in CD include limiting and/or reversing the endothelial-to-mesenchymal transition, which is a major pathology of fistulas [17,18,19].

PAs are a great tool for potential modification of polymers because of the carboxylic acid functional groups. Recently, our group developed a biodegradable polyurethane-based shape memory polymer (SMP) hydrogel foam that could be used for fistula filling and healing [20]. SMPs are materials that are synthesized in a primary shape and can be deformed and stored in a temporary, secondary shape. Upon application of a stimulus, such as exposure to body temperature water, SMPs return to their primary shape. Shape memory polyurethanes have been employed in biomedical applications for over 20 years due to their high tunability and biocompatibility [21,22,23,24]. More recent work explored the use of SMP hydrogels for applications in sensors and tissue engineering [25,26,27]. The hydrogel foams that we designed can be compressed radially and stored in a temporary low-profile geometry that allows for facile implantation into a fistula site. Upon exposure to water in the body, the foams can radially expand to fill the fistula tract. The shape memory properties are based on hydrogen bonds between urethane linkages, polyvinyl alcohol (PVA), and/or cornstarch molecules in the polymer backbone. These materials can be easily modified due to the large number of free hydroxyl groups present in both the PVA and cornstarch. 

One method for PA incorporation into these materials involves using isocyanate reactions that are commonly employed in polyurethane synthesis. PAs were previously incorporated into polyurethane-based materials using one-pot methods in applications for hemorrhage control [28,29]. By reacting the carboxylic acid on PAs with a diisocyanate, an amide linkage is formed. The free isocyanate end can then be reacted with free hydroxyls on PVA or cornstarch, forming a polymer with pendant PAs. Alternatively, PAs can be physically incorporated into the hydrogels using hydrogen bonds between OH groups, allowing for simpler synthesis and faster release into the surrounding environment. 

Here, we employed chemical and physical incorporation approaches to modify crosslinked PVA-based polyurethane hydrogels with three cinnamic acid-based PAs with varied side groups: (cinnamic (CA), p-coumaric (PCA), and caffeic (Ca-A) acid). We evaluated the effects of modification on mechanical and shape memory properties, cytocompatibility, PA release, and antimicrobial efficacy. The shape memory properties of these materials would allow for rapid fistula filling while PAs provide local antimicrobial properties. Additionally, the inclusion of PAs can be used to tune intermolecular interactions between polymer chains to alter material mechanical and shape memory properties. 

## 2. Methods

### 2.1. Materials

Hexamethylene diisocyanate (HDI), cinnamic acid (CA), p-coumaric acid (PCA), and caffeic acid (Ca-A) were purchased from TCI chemicals (Portland, OR, USA). Polyvinyl alcohol (PVA, 25,000 MW) was purchased from Polysciences (Warrington, PA, USA). Dimethyl sulfoxide (DMSO) and phosphate buffered saline (PBS) tablets were purchased from Fisher Scientific (Waltham, MA, USA).

### 2.2. Synthesis

#### 2.2.1. Chemically Incorporated PA-PVA Films

**Figure 1:**
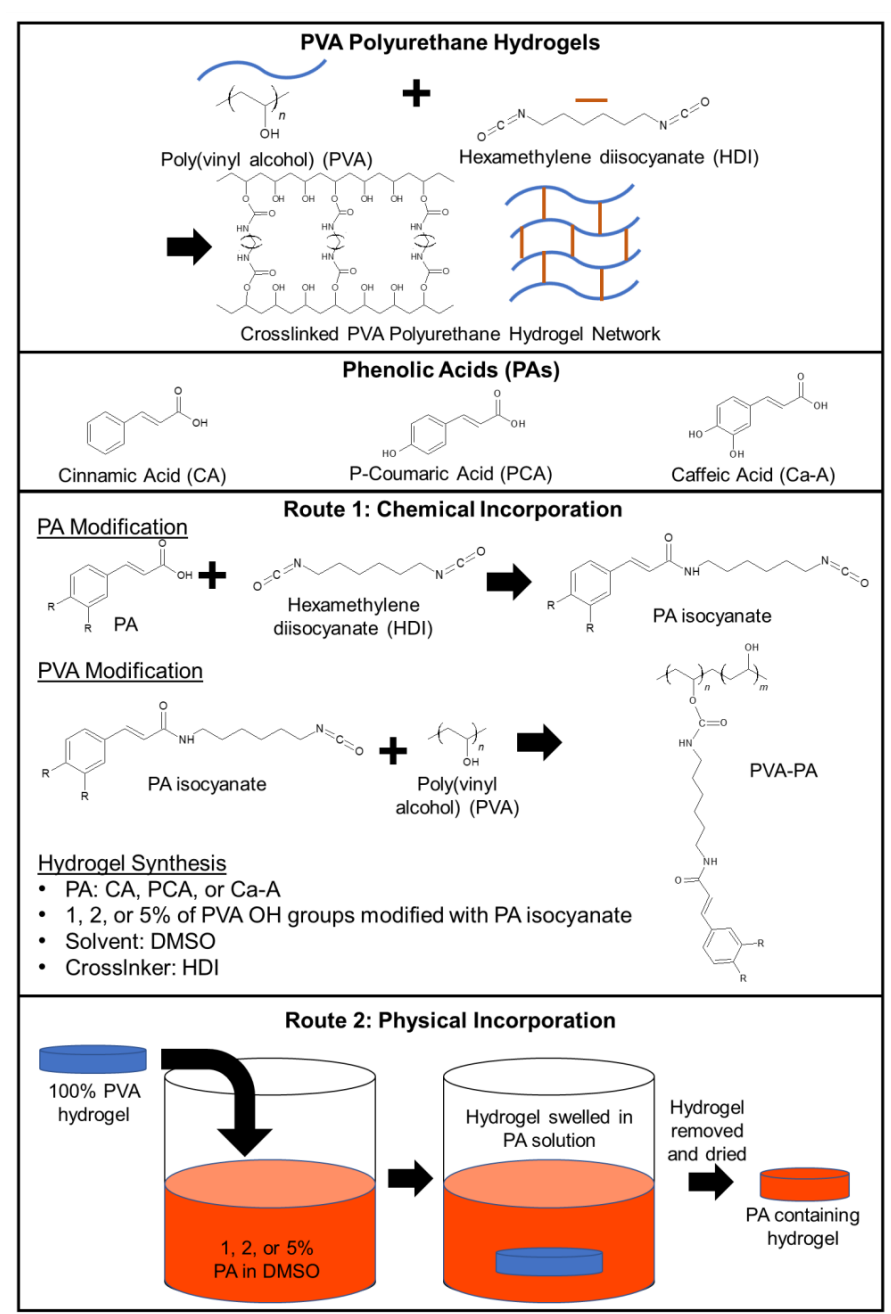
Schematic representation of routes of chemical and physical incorporation of phenolic acids into crosslinked PVA polyurethane hydrogels.

PA-modified films were made by first reacting PAs with hexamethylene diisocyanate (HDI) in a 1:1 molar ratio in DMSO to form PA isocyanates, Figure 1. PAs (CA, PCA, and Ca-A) were dissolved in sieve-dried DMSO in a Flacktek speed mixer cup. HDI was then added inside a moisture-controlled glovebox (Labconco) to react in a 1:1 molar ratio with the PAs. The cup contents were mixed using a Flacktek high-speed mixer at 3500 rpm for 15 s and placed in a 50 °C oven to react for 24 h. Reaction contents were kept sealed to minimize exposure to atmospheric water and potential side reaction of isocyanates. PA isocyanate synthesis was tracked using Fourier transform infrared (FTIR) spectroscopy based on the reduction of the isocyanate peak at ~2250 cm^−1^. Once the reduction stabilized, the reaction was considered complete.

PVA was then dissolved in sieve-dried DMSO in a round bottom flask at 90 °C at 100 mg/mL under nitrogen. When completely dissolved, the solution was cooled to room temperature while maintaining atmospheric nitrogen to minimize water exposure. Unmodified HDI was added as a crosslinker (0.033 mol. eq. relative to PVA OH) to the PA isocyanates (1, 2, or 5 mol. eq. relative to PVA OH) in the moisture-controlled glovebox. The cup was placed in a high-speed mixer at 3500 rpm for 15 s. Dissolved PVA was then added to the speed mixer cup while inside of the glovebox and mixed for an additional 15 s at 3500 rpm. The cup contents were poured into a glass petri dish inside the glovebox, sealed to prevent exposure to atmospheric water, and allowed to cure overnight at 50 °C. Resulting hydrogel films were washed twice in deionized (DI) water for 20 min each to remove residual DMSO. Samples were dried in a 50 °C vacuum oven for 24 h and, upon completion of drying, were examined with FTIR spectroscopy. DMSO removal was confirmed based on final dry weights relative to theoretical polymer content per volume.

#### 2.2.2. Physically Incorporated PA-PVA Films

PVA was dissolved in sieve-dried DMSO at 90 °C at 100 mg/mL under nitrogen. The mixture was cooled to room temperature while sealed to maintain dry conditions. HDI was added to the PVA in the moisture-controlled glovebox and the solution was placed in the high-speed mixer at 3500 rpm for 10 s. Contents were poured into a glass petri dish, sealed, and cured overnight at 50 °C. Films were washed twice in DI water for 20 min each. PA solutions were prepared at 1, 2, and 5 wt% in DMSO. PVA film samples (6 mm biopsy punches and tensile dog bones) were incubated in these solutions at 50 °C for 24 h, Figure 1. Following physical incorporation, sample pieces were washed with water and dried in the 50 °C vacuum oven to remove DMSO before characterization.

### 2.3. Characterization

#### 2.3.1. Gel Fraction

Gel fractions were measured on chemically incorporated PA-PVA hydrogels only, as PAs would be released from physically incorporated PA-PVA hydrogels, altering the results. Before washing films with DI water, 6 mm biopsy punches were taken (*n* = 3). Samples were dried in a 50 °C vacuum oven for 24 h. Upon drying, initial weight (W_i_) was obtained. Samples were placed in a vial and swollen in DMSO. Vials were incubated at 50 °C for 24 h. DMSO was removed and samples were vacuum dried at 50 °C for 24 h. Film pieces were reweighed to obtain dry weight (W_d_). Gel fraction was calculated as:

$$Gel\;fraction\left(\%\right)=\frac{W_{d}}{W_{i}}*100$$

#### 2.3.2. Swelling

Swelling ratios were obtained on chemically incorporated PA-PVA hydrogels. Following the recording of the dry weight (W_d_), samples were placed in vials and swelled in 37 °C deionized (DI) water. Vials were placed on a 37 °C orbital shaker to maintain temperature for 24 h. Samples were removed from the vial and weighed (W_s_). The swelling ratio was calculated as:

$$Swelling\;ratio=\frac{W_{s}-W_{d}}{W_{d}}$$

#### 2.3.3. Physically Incorporated PA Content

Dried PVA hydrogels were weighed (W_c_) before physical incorporation (*n* = 3). After the completion of physical incorporation, hydrogels were dried under vacuum and re-weighed (W_L_). PA content, as a percent of the hydrogel mass, was calculated as:

$$PA\;content\left(\%\right)=\frac{W_{L}-W_{C}}{W_{c}}*100$$

#### 2.3.4. Mechanical Properties

Film samples for both chemically (*n* = 3) and physically (*n* = 3) incorporated gels were characterized in terms of tensile properties. Dog bone specimens were placed in a vial of DI water at 37 °C for 1 h to hydrate before testing. Dog bones had a length of 6.25 mm and a width of 1.5 mm. Samples were stretched at a rate of 5 mm/minute until failure. Resulting stress/strain curves were utilized to determine tensile modulus (slope of curve in linear region) and elongation at break (strain at failure). 

#### 2.3.5. Release Rates

Release rates of PAs from chemically (*n* = 3) and physically (*n* = 3) incorporated hydrogels were examined using UV-vis spectroscopy (Evolution 60, Thermo Scientific, Waltham, MA, USA). Calibration curves for CA, PCA, and Ca-A were made using a serial dilution of each PA in a 1:1 mixture of DMSO:PBS at wavelengths of 270, 280, and 290 nm, respectively. Biopsy punches (6 mm) were placed in 2 mL centrifuge tubes. Samples were submerged in PBS at 37 °C, and media was changed at 1 h, 1 day, 7 days, 14 days, and 28 days. At these time points, media was transferred to another 2 mL centrifuge tube, diluted by 2 in DMSO to provide a 1:1 DMSO:PBS solution, and characterized using UV-vis to test release rates. Measurements were calculated as both total PA released and % release relative to initial PA loading after normalizing to a blank consisting of 1:1 DMSO:PBS. 

### 2.4. Cell Viability and Growth

All cell viability testing was completed using human colon (Caco-2) cells (ATCC, Manassas, VA, USA) incubated with physically and chemically incorporated samples (*n* = 3). Cells were cultured in DMEM supplemented with 10% fetal bovine serum and 1% penicillin–streptomycin (pen-strep). Cells were trypsinized and placed into a 24-well plate at a density of 5000 cells per well. Transwell inserts containing 6-mm diameter cylindrical samples were then placed into testing wells. Cell viability was assessed at 24 h using a resazurin assay (Alamar Blue) and fluorescence absorbances (excitation 530 nm and emission 590 nm) were measured by a plate reader (BioTek Synergy 2). Cell growth was assessed in CA and PCA gels over 7 days, also using a resazurin assay. Ca-A hydrogels were not assessed for growth due to poor initial cytocompatibility. Cell viability was calculated as:

$$Cell\;Viability\left(\%\right)=\frac{{Abs}_{Sample}-{Abs}_{Blank}}{{Abs}_{Control}-{Abs}_{Blank}}\times 100$$

where blanks contained media only, and the control contained cells with media. Cell growth was examined by comparing the relative absorbance values at 7 days to the initial absorbance after 24 h. Relative absorbance was calculated as:

$$Relative\;absorbance=\frac{{Abs}_{Sample\;day7}-{Abs}_{Blank}}{{Abs}_{sample\;day1}-{Abs}_{Blank}}$$

### 2.5. Antimicrobial Properties

Hydrogel samples (*n* = 3) were cut into 6 mm diameter disks and UV sterilized for 20 min on each side. Antimicrobial properties were examined in *Escherichia coli* (*E. coli*). Bacteria were grown in 5 mL of lysogeny broth (LB) at 37 °C overnight. After 16 h, 1 mL of the bacterial solution was diluted to 10 mL with LB. The solutions were then incubated until they reached an optical density of 0.6, which was confirmed by plate reader. Samples were placed into a 96-well plate and 100 μL of the bacterial solution was placed into each well. The plate was then incubated for 1 h at 37 °C. The solution in the well was diluted by a factor of 10^7^ in LB, and 10 μL of the bacterial solution from each well was drop-plated onto an LB-agar plate and incubated for 18 h at 37 °C [30]. Images of the plates were taken at the completion of the incubation and examined for qualitative analysis of colony forming unit (CFU) density based on whether the droplet areas were completely covered with a ‘lawn’ of bacteria or individual colonies could be distinguished.

### 2.6. Biofilm Formation

The effects of chemically and physically incorporated CA and PCA on biofilm formation were examined using *Staphylococcus aureus* (*S. aureus*). Overnight cultures of *S. aureus* were initially prepared in a similar way to that described for *E. coli* in Section 2.5. The bacterial solution was grown to an optical density of 0.4. Samples were placed into a 12-well plate with 2 mL of LB with 0.25% glucose. Then 400 μL of the bacterial solution was added and incubated for 24 h under static conditions. The samples were then prepared for either scanning electron microscopy (SEM, Jeol NeoScope JCM-5000, Peabody, MA, USA) imaging of the attached biofilm (*n* = 3) or crystal violet (CV) staining of the surrounding biofilm (*n* = 3). For SEM imaging, the samples were washed 3 times with DI water and 3 times with 0.85% NaCl and then fixed using 2.5% glutaraldehyde for 1 h. The samples were placed in a series of solutions with increasing acetone concentrations (15, 30, 50, 60, 75, and 100% in water) for 15 min each to dehydrate. The samples were air-dried overnight and imaged via SEM at 1500 magnification and 15 kV. 

For CV staining, samples were carefully removed from wells and 400 μL of 0.1% CV was placed into the wells for 20 min. The well was then washed 3 times with DI water. The CV was redissolved by adding 30% acetic acid to each well. The solution was then read at an absorbance of 540 nm by a plate reader. Relative absorbance was calculated as:

$$Relative\;absorbance=\frac{{Abs}_{Sample}}{{Abs}_{Control}}$$

The control sample was a non-modified PVA film.

### 2.7. Shape Memory Properties

All hydrogel formulations containing CA and PCA were tested for shape memory. Ca-A was excluded due to poor cytocompatibility and antimicrobial properties. For chemically incorporated hydrogels, the samples were allowed to incubate in DI water overnight at 37 °C. Films were then punched with a dog bone cutter, folded, and placed with the folded ends facing down into the single well of a 96-well plate. Placement into the well plate kept the samples from unfolding while drying. The samples were vacuum dried for 48 h to fix the secondary shape. Physically incorporated dog bone-shaped hydrogels were prepared as mentioned in Section 2.2.2, but prior to drying they were folded and placed end-down into a 48-well plate. Larger-well plates were used due to higher swelling and the more brittle nature of the hydrogels when swollen in DMSO during PA incorporation. After drying, samples were removed from wells and incubated for 24 h at ambient humidity and room temperature. Shape fixity was assessed by tracking the change in the distance between the two folded ends and calculated as:

$$Shape\;fixity\left(\%\right)=100-(\frac{{Distance}_{24hour}-{Distance}_{initial}}{{Distance}_{initial}}\times 100)$$

Samples were then placed in 37 °C water for 30 min and imaged to qualitatively assess shape recovery. 

### 2.8. Statistical Analysis

Measurements are presented as mean ± standard deviation. Single factor ANOVA with Tukey’s post hoc test were used determine statistical significance. A *p*-value of <0.05 was taken as statistically significant.

## 3. Results

### 3.1. Characterization

#### 3.1.1. Gel Fraction and Swelling Ratio

Gel fractions for chemically incorporated PA-PVA hydrogel formulations ranged from 77–100%, Table 1, which is indicative of successful crosslinking. In general, hydrogels containing Ca-A had the lowest gel fractions, ranging between 77 and 85%, compared to the highest gel fractions in CA-incorporated PVA hydrogels, which were between 94 and 100%. Swelling ratios were all at least 50% higher than the initial dry weight, and PA incorporation reduced swelling in comparison with the control. In CA hydrogels, swelling was highest in the 2% gels. In PCA hydrogels, swelling was similar in 1% and 2% gels and lowest in 5% gels. In Ca-A hydrogels, swelling was similar in 1% and 2% gels and highest in 5% gels. 

#### 3.1.2. Surface Chemistry

**Figure 2:**
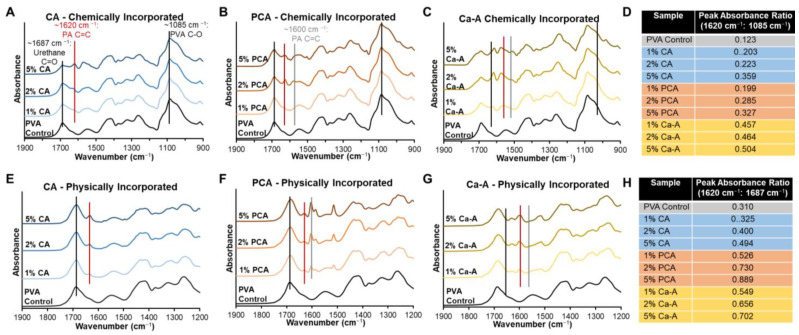
Fourier transform infrared spectroscopy (FTIR) of (**A**–**C**) chemically incorporated CA, PCA, and Ca-A-PVA hydrogels and (**E**–**G**) chemically incorporated CA, PCA, and Ca-A-PVA hydrogels, with peak absorbance ratios for key peaks indicative of phenolic acid (PA) (**D**) chemical and (**H**) physical incorporation. Black lines indicate the C=O of the urethane and the C-O of PVA that are present in all spectra. Red and grey lines indicate C=C linkages in incorporated PA rings.

For chemical characterization of crosslinked, 3-dimensional networks, FTIR enables analysis of surface chemistry to compare effects of selected modifications. The FTIR spectra of synthesized hydrogels are shown in Figure 2. A peak at ~1687 cm^−1^ is present in all formulations, representing the carbonyl of the urethane in the polymer backbone, and the C-O peak of PVA’s backbone is visible at ~1085 cm^−1^ (black lines). In all PA-containing hydrogels, peaks associated with alkene bonds in the PA ring structures at ~1600 and 1620 cm^−1^ (grey and red lines, respectively) increase with increased PA content. As PA content increases in both chemically and physically incorporated hydrogels, the absorbance ratios between PA ring peaks at ~1620 cm^−1^ and control peaks at ~1085 cm^−1^ and ~1687 cm^−1^, respectively, increases, Figure 2D,H. 

#### 3.1.3. Physically Incorporated PA Content

All physically incorporated PA-PVA hydrogels had an increase in PA absorption as the PA concentration increased from 1–5% (Table 1). CA showed the lowest incorporation percentage, with all CA formulations absorbing under 50% of the polymer weight in CA. PCA had the highest incorporation amount at all three PA solution concentrations. Incubation of samples in the 5% PCA and Ca-A solutions resulted in over 100% absorption of PAs relative to the polymer weight, with PCA having the greatest absorption, at 144% of the polymer weight. 

#### 3.1.4. Mechanical Properties

**Figure 3:**
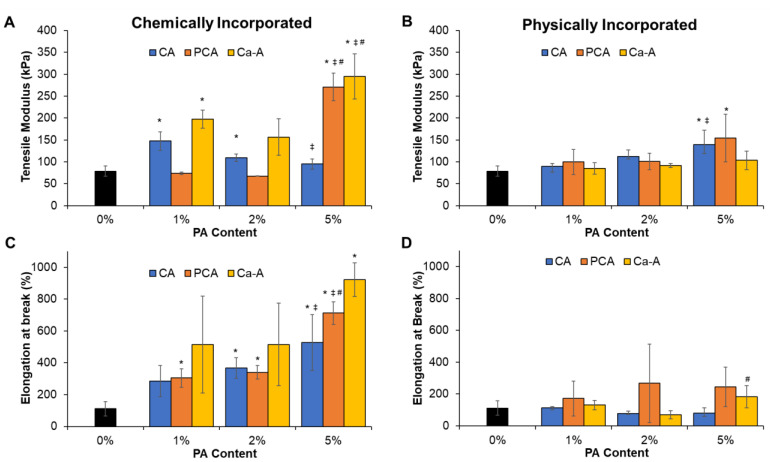
Chemically incorporated PA-PVA hydrogel (**A**) tensile modulus and (**B**) elongation at break in the wet state. Physically incorporated PA-PVA hydrogel (**C**) tensile modulus and (**D**) elongation at break in the wet state. *n* = 5; mean ± standard deviation displayed. Symbols indicate *p* < 0.05 relative to control (*), 1% (^‡^), and/or 2% (^#^) PA hydrogels.

All PA-PVA hydrogels had a similar or higher modulus than the control of 79 kPa, Figure 3A,B. As the chemically incorporated CA content increased, the modulus decreased to values that were comparable with that of the control, Figure 3A. Chemically incorporated PCA hydrogels had constant moduli at 1 and 2% PCA, and a large increase in modulus (3.7-4X) was observed in 5% PCA gels. Chemically incorporated Ca-A hydrogels had the highest modulus at each concentration, with similar stiffnesses at 1 and 2% Ca-A content and increased stiffness at 5% Ca-A. Elongation at break was higher in chemically incorporated PA hydrogels than in the PVA control and in corrollary physically incorporated PA hydrogels. Generally, as the PA content and the number of pendant hydroxyls on chemically incorporated PAs increased, the elongation at break increased, Figure 3C.

In general, as the PA content was increased in physically incorporated PVA hydrogels, the modulus increased, Figure 3B. Hydrogels with physically incorporated CA and PCA that contain 0–1 hydroxyl groups on the PA ring had larger increases in modulus as PA content increased. Physical incorporation of PAs had reduced effects on elongation at break, and several formulations had highly variable elongation at break after PA incorporation. Higher concentrations of physically incorporated CA (2 and 5%) resulted in lower elongation at break compared with the control (no statistical difference), Figure 3D. PCA gels had the highest elongation at break within the physically incorporated PA hydrogels, with an increase between 1 and 2% PCA and a plateau between 2 and 5% PCA, but elongation measurements were highly variable. A high concentration of Ca-A (5%) also increased elongation at break compared with control. 

#### 3.1.5. PA Release Rates

**Figure 4:**
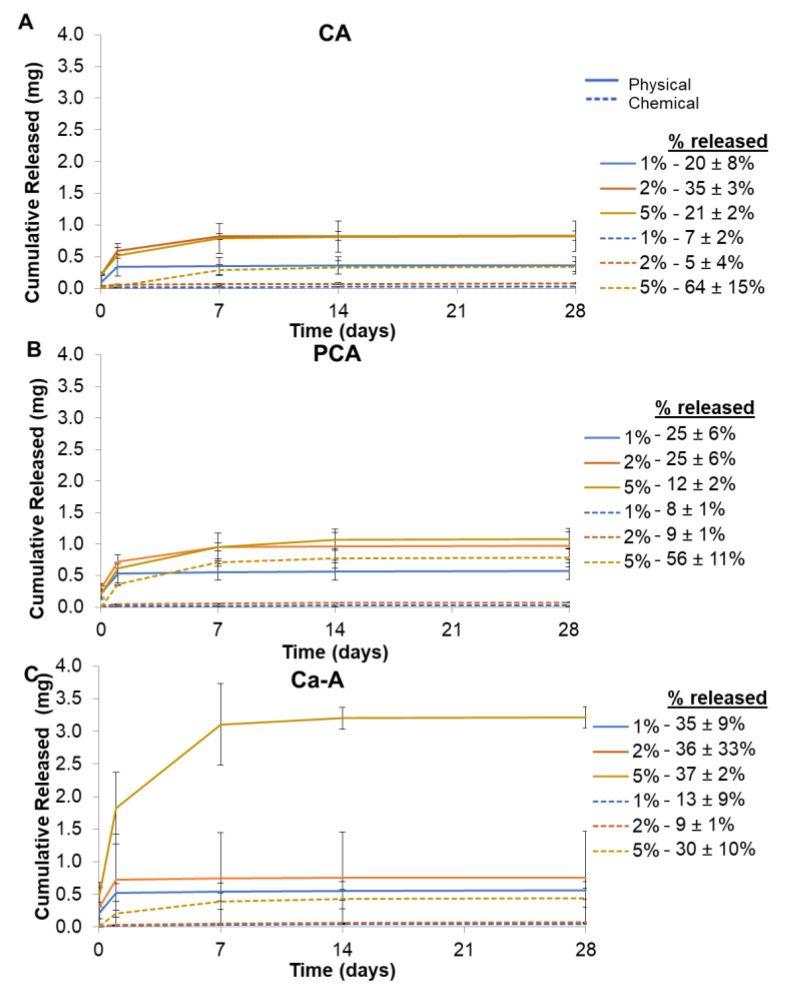
Release of physically and chemically incorporated PAs from PVA hydrogels with (**A**) cinnamic acid, (**B**) *p*-coumaric acid, and (**C**) caffeic acid. Percent of PA content released at 28 days is provided in legends. *n* = 3; mean ± standard deviation displayed.

Hydrogels with physically incorporated PAs had higher, sustained PA release in PBS at 37 °C compared to chemically incorporated PAs, Figure 4, solid lines. All formulations showed a burst release of PA at 1 day. As PA content increased from 1 to 5%, PA release increased, and the point at which release plateaued was delayed from 1 day for 1% PA incorporation to 7–14 days for 5% PA incorporation. Thus, release was sustained for longer time frames in higher-PA-content gels. Physically incorporated Ca-A hydrogels showed the largest released PA content (35–37%) at all concentrations of PA loading. In general, there were relatively low levels of release in the chemically incorporated PA-PVA hydrogels, Figure 4, dashed lines. Hydrogels with 5% PA content had the highest release amounts. Minimal PA release was observed after 14 days in the majority of physically and chemically incorporated formulations. 

### 3.2. Cell Viability

**Figure 5:**
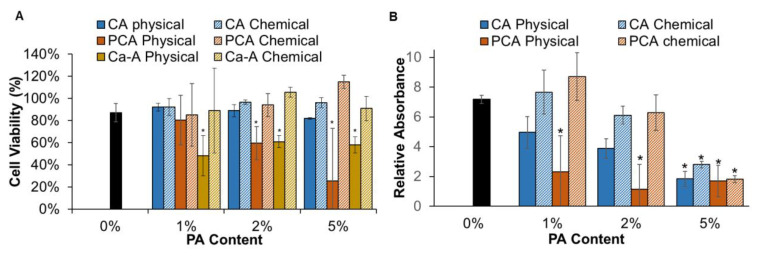
(**A**) Caco-2 cell viability after 24 h of incubation with all hydrogel formulations, and (**B**) cell growth based on relative absorbance (compared with cells at 24 h) after 7-day incubation with CA and PCA hydrogels. *n* = 3; mean ± standard deviation displayed. * *p* < 0.05 relative to PVA control.

As an initial indication of cytocompatibility, Caco-2 cell viability was assessed after 24 h of indirect incubation with hydrogels, Figure 5A. The control PVA polyurethane hydrogels (black bars) had high cytocompatibility, enabling characterization of the effects of incorporated PA release on scaffold cell interactions. Chemically incorporated hydrogels generally had greater than 75% cell viability (based on ISO 10993-5 benchmark) [31], except for 2 and 5% Ca-A formulations. For physically incorporated hydrogels, decreased cell viability was observed with increased CA and PCA content, with all physically incorporated CA hydrogels having >75% cell viability and only 1% PCA having viability >75% at 24 h. Hydrogels with physically incorporated Ca-A all showed lower cell viability of 48, 61, and 58% for 1, 2, and 5% Ca-A, respectively. 

To further characterize promising formulations, Caco-2 growth was measured over a week in the presence of CA and PCA-containing hydrogels. In terms of cell growth over 7 days (relative to cell numbers at 24 h of incubation with each sample), all tested hydrogels promoted increases in cell numbers over time. However, physically incorporated PCA resulted in the slowest cell growth, Figure 5B. Cell growth decreased with increasing concentration of CA, with 1% CA having the lowest impact on cell growth. Chemically incorporated PAs only showed significant effects on cell growth relative to the control hydrogel at 5% modification. 

### 3.3. Antimicrobial Properties

#### 3.3.1. Colony Forming Units (CFUs) of *E. coli* (1 h Incubation)

**Figure 6:**
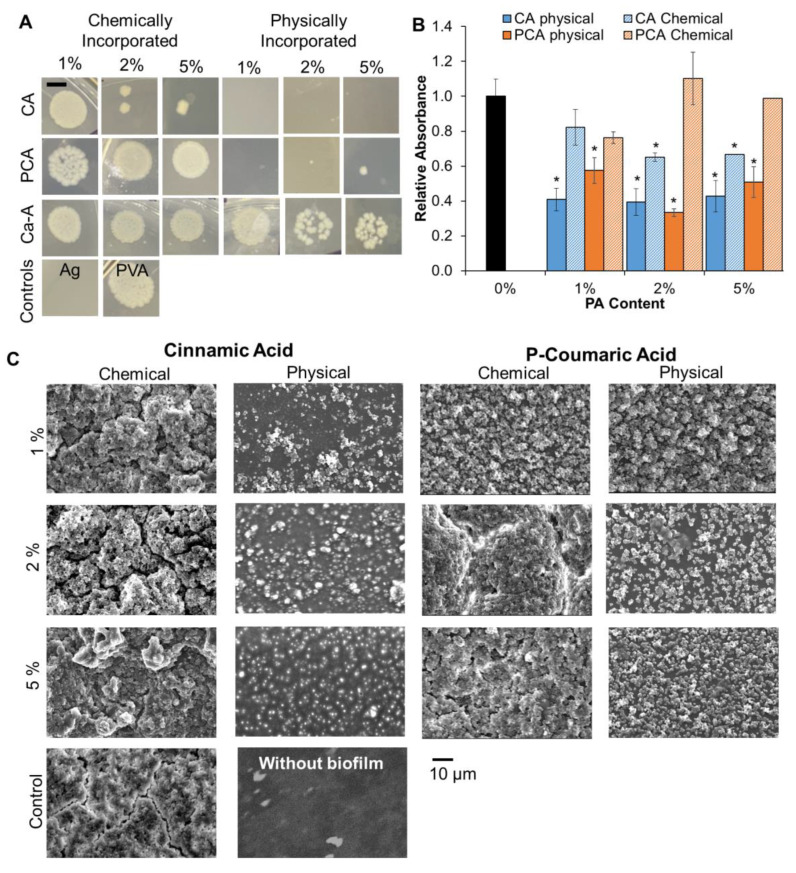
(**A**) Representative images of *E. coli* colony forming unit density after incubation with chemically and physically incorporated PA hydrogels. Scale bar of 5 mm applies to all images. (**B**) Crystal violet biofilm staining of *S. aureus* in wells surrounding physically and chemically incorporated CA and PCA films. *n* = 3; mean ± standard deviation displayed, * *p* < 0.05 relative to control PVA hydrogel (0% PA) in LB media. (**C**) SEM images of *S. aureus* biofilms on CA, PCA, and control hydrogels. Scale bar applies to all images.

Pathogenic *E. coli* is commonly found in the intestinal tract and is associated with Crohn’s fistulas [8]. Further research suggests that *E. coli* could contribute to triggering fistula formation by inducing the epithelial-to-mesenchymal transition that is consistently observed in Crohn’s fistulas [32]. Thus, antimicrobial activity against *E. coli* was assessed first. None of the chemically incorporated PCA or Ca-A hydrogels showed qualitative effects on *E. coli* CFU density in comparison with the PVA control after 1 h of incubation, Figure 6A. A ‘lawn’ of bacteria can be viewed over the full sample droplet areas, with no visible individual colonies. Chemically incorporated CA at 2 and 5% reduced CFU density considerably to a point where individual colonies could be seen (vs. ‘lawn’ of *E. coli* in controls, PCA, and Ca-A). Physically incorporated CA hydrogel incubation resulted in no visible CFUs at all concentrations (clean droplet areas), showing improved antimicrobial properties to levels that were comparable to the silver-based wound dressing clinical control (Ag). Similarly, physically incorporated PCA hydrogels had little to no CFU formation. Low concentrations of physically incorporated Ca-A (1%) did not visibly affect CFU density, again resulting in ‘lawn’ formation. Higher concentrations of physically incorporated Ca-A reduced bacterial density to levels where individual colonies could be viewed, but still had relatively high CFU densities. 

#### 3.3.2. *Staph. aureus* Biofilm Formation (24-h Incubation)

*S. aureus* is identified as one of the most important wound pathogens, and prior research found that perianal fistulas are primarily colonized by gram positive pathogens, such as Staphylococcus [33]. Thus, *S. aureus* biofilm formation was analyzed in the surrounding media and on the surface of CA and PCA hydrogels. Visual assessment of biofilm by SEM showed decreased biofilm formation on physically incorporated PCA and CA hydrogel surfaces, Figure 6C. Chemically incorporated PAs showed limited effects on biofilm formation in comparison with controls. Crystal violet staining of biofilm in the surrounding well confirmed significant decreases in biofilm formation compared with the control in physically incorporated CA and PCA hydrogels, Figure 6B. All physically incorporated CA hydrogels showed comparably low biofilm formation in surrounding media (0.40, 0.39, and 0.42 relative absorbance compared with PVA hydrogel control in 1, 2, and 5% CA, respectively). Hydrogels with 2% physically incorporated PCA had the best biofilm inhibition (0.33 relative absorbance), and 1 and 5% PCA showed 0.57 and 0.50 relative absorbance, respectively. In the chemically modified hydrogels, only 5% CA showed a significant reduction in biofilm formation compared with the control, and the relative reduction was lower than that of 5% physically incorporated CA. 

### 3.4. Shape Memory Properties

**Figure 7:**
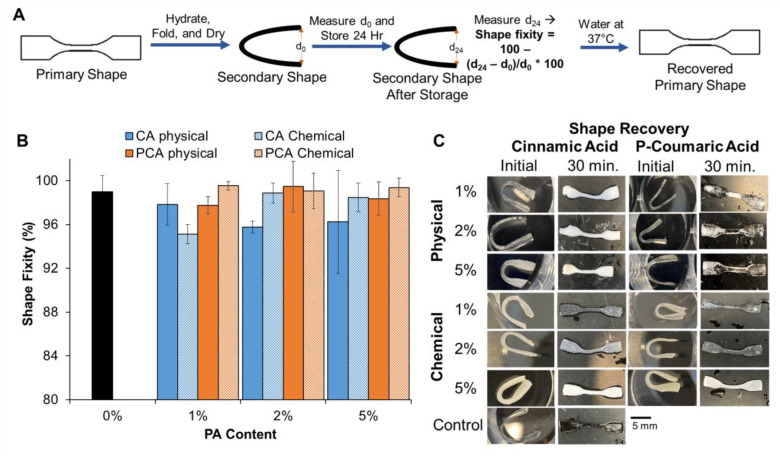
Shape memory properties of CA and PCA hydrogels. (**A**) Schematic representation of process used to fix secondary shape, measure shape fixity, and induce shape recovery. (**B**) Shape fixity as measured by the change in distance between folded sample edges after 24 h of storage. *n* = 3; mean ± standard deviation displayed. (**C**) Complete (100%) shape recovery (unfolding) after incubation in 37 °C water for 30 min. Scale bar applies to all images.

Based on the promising antimicrobial and cytocompatibility properties of CA and PCA hydrogels, their shape fixity and recovery were characterized to evaluate shape memory properties, Figure 7A. Some small shape changes were observed in samples that were fixed into a folded secondary shape after 24 h of storage at ambient humidity and room temperature; however, all average changes were less than 5% of the original length (measured as distance between folded ends), indicating high shape fixity in these materials, Figure 7B. All tested hydrogels showed the ability to recover to their primary, unfolded state after 30 min in 37 °C water, Figure 7C. The observed color changes in hydrogels in the images in Figure 7C are due to water absorption during the shape recovery process. 

## 4. Discussion

The gel fraction, FTIR, and PA absorption data all provide evidence of successful chemical (gel fraction and FTIR) and physical (FTIR and PA absorption) PA incorporation into PVA hydrogels. For chemically incorporated hydrogels, the gel fraction depends on both PA concentration and the number of phenolic hydroxyls. In general, gel fraction decreased in all formulations with PAs that have hydroxyl substituted rings (i.e., PCA and Ca-A). Specifically, CA, which does not have any phenolic hydroxyls, had the highest gel fractions at all concentrations, whereas Ca-A has the highest number of pendant hydroxyls (2) and had the lowest gel fractions. This result may be attributed to reactions between phenolic hydroxyls on PCA and Ca-A and isocyanates, which would consume free isocyanate ends to limit crosslinking and lower gel fractions. The phenolic hydroxyls are less reactive than primary and secondary aliphatic alcohols [34]. Thus, the reaction of the carboxylic acid end of PAs should be more efficient; however, it is possible that some undesired reactions occurred between the phenolic rings during incorporation. To improve gel fractions in future work, other methods of chemical incorporation may be employed, such as esterification of PA carboxylic acids with a separate monomer to provide hydroxyl or amine end groups with higher isocyanate reactivity [29,35,36].

Physical PA incorporation amount was dependent on the number of hydroxyls and the concentration of PAs in the swelling solution. At concentrations of 1%, the amount of PA absorbed into the material was between 24 and 35% of the polymer weight. This concentration of PAs is significant, as concentrations as low as 270 µM show antimicrobial efficacy [37]. Even at low concentrations, the loading shows dependence on the hydroxyl content of the PA, where PAs with increased hydroxyl content had higher loading efficiencies. This trend becomes more evident in hydrogels swollen in higher PA concentrations. At 5%, the PA content of PCA and Ca-A hydrogels was three times that of CA gels. The hydroxyls allow for an increased number of intermolecular interactions between PVA chains and the physically incorporated PAs. Namely, increasing the number of hydrogen bonding sites enables more efficient incorporation of PCA and Ca-A. Phenolic compounds were incorporated into physically crosslinked PVA hydrogels in prior work. These materials had an increased glass transition temperature, which was similarly attributed to the physical crosslinks created by hydroxyls on the phenolic compounds [38]. 

The addition of PAs both physically and chemically leads to changes in the material properties of the hydrogels. First, chemical incorporation affected hydrogel swelling. PA-modified PVA hydrogels had decreased swelling in all formulations compared to the control PVA hydrogel. CA incorporation into the hydrogels led to the largest decreases in swelling; this effect is attributed to its relatively hydrophobic ring with no hydroxyls, which limits water interactions with the resulting hydrogels. A previous study showed that increased modification of chitosan with relatively hydrophobic tyramine led to decreased swelling [39]. The modification of PVA with both CA and HDI increases the hydrophobic regions of the polymer by the addition of 6-carbon chains in HDI and a benzene ring in CA to produce a similar effect on swelling. PAs with hydrophilic hydroxyl-substituted rings (PCA and Ca-A) showed reduced effects on swelling properties. Further examination of the effects of chemical modification concentrations is required in future work, as no clear trends were observed between swelling ratios and PA concentrations. 

The effect of PA incorporation on mechanical properties was examined by tensile testing. Physical incorporation of PAs into hydrogels increased tensile modulus and affected ultimate elongation. General trends of increased modulus were observed with decreasing PA hydroxyl substitutions and/or increasing PA content. CA showed the largest effect on modulus at 1 and 2%, and PCA showed a similar modulus to that of CA at 5% incorporation. Larger amounts of chemically incorporated PAs (5%) increased modulus in all formulations. At lower concentrations, increased modulus was seen in CA and Ca-A, but not PCA. Hydrogels modified with CA result in a pendent hydroxyl on the PVA chain being substituted with a benzene ring from CA. The incorporation of benzene rings into hydrogels can increase hydrogel moduli due to the bulky and restricted structure [40,41,42]. In contrast, PCA seems to have a plasticizing effect at lower concentrations, separating chains to reduce intermolecular bonds, and decreasing the modulus in comparison with the control. This effect is similar to data previously published on poly(caprolactone)-based polymers with chemically incorporated PCA, which showed decreased modulus and increased elongation at break [43]. 

Improved elongation at break was seen in all chemically modified hydrogels and, to some extent, in physically modified hydrogels with PCA (all concentrations) and Ca-A (1 and 5%). Chemical modification of PVA-based hydrogels previously improved elongation at the break due to a decrease in the order of crystalline regions because of the disruption of highly regular hydroxyl repeats [44]. Physical incorporation of CA decreased elongation, with increased effects as CA content increased, which matches the observed increases in stiffness with increased physically incorporated CA. Conversely, higher concentrations of physically incorporated PCA and Ca-A generally increased elongation at break, although larger variations were observed in the elongation measurements. 

Depending on their location, fistula tracts can traverse multiple tissue types, including intestine, colon, rectal, fat, muscle, and/or skin. Perianal fistulas are most common, and primarily affect colon/rectal tissues and skin. Colon tissue has a reported Young’s modulus of ~300–400 kPa [45], whereas skin has a modulus of ~100–200 kPa [46]. Human colon and rectal tissues have a reported elongation at break of ~180–300% [47,48], and human skin ultimate elongation is ~120–300% [49]. Thus, incorporation of PAs into PVA hydrogels enables improved matching of both stiffness and elongation to surrounding tissues in Crohn’s fistulas. Beyond matching native tissue properties, elasticity and modulus are important factors to consider for Crohn’s fistula filling due to the peristaltic environment of the gastrointestinal tract. For example, fibrin glue, which has a Young’s modulus of ~12 kPa and an ultimate elongation of ~100%, is susceptible to dislodgement in Crohn’s fistulas [50,51]. Although these hydrogels should be characterized in physiologically relevant models of fistulas, increasing stiffness of fistula filling materials may increase filler stability in the fistula tract to improve outcomes. On a fundamental materials science level, modulus (stiffness) is typically inversely related to elongation, and these properties are difficult to tune independently. Incorporation of PAs provides a new tool to increase both properties simultaneously, which could be valuable in a range of materials applications.

In general, release rates of PAs were highest in physically incorporated hydrogels. All physically incorporated PAs showed a burst release over 24 h, and release after 24 h was dependent on PA concentration. PA release also correlated with loading efficiency, with higher release from hydrogels that had larger amounts of physically incorporated PA (and higher numbers of hydrogen bonds). Lower PA release was measured in chemically incorporated hydrogels. The amide linkage that forms between HDI and the PAs is non-degradable in a PBS-based solution. In general, the formulations with lower gel fractions showed higher PA release. Thus, by increasing gel fractions, the release of chemically incorporated PAs could be better predicted. Additionally, modifications in hydrogel washing could reduce unwanted PA release in future work. Owing to the low solubility of PAs in water, removal of unreacted monomer may be higher in other solvents, such as DMSO. 

Chemically incorporated PA hydrogels showed good cytocompatibility for all compositions. Cytocompatibility of physically incorporated hydrogels was reduced with increased PA concentration and increased PA ring hydroxyls, which correlates with the PA release data. CA showed the best cytocompatibility at 24 h, with all CA concentrations having cytocompatibility above the 75% benchmark determined by ISO 10933-5 [52]. PCA was only cytocompatible at 1%, and PCA hydrogels generally showed the largest variability in cell testing. This result may be attributed to the ability of PCA to affect cell attachment [53]. Ca-A materials all showed cell viability below the ISO standard, which is consistent with previous reports [16,54]. This effect was observed even in the 1% Ca-A gels, which had a low PA release compared with the CA and PCA hydrogels, indicating that Ca-A has higher potential toxicity than the other tested PAs. Owing to the cytotoxic nature of Ca-A, it was not examined for cell growth over 7 days. Both CA and PCA affected surrounding cell growth over 7 days. Increasing PA concentration in the hydrogels slowed cellular growth. CA hydrogels showed superior growth in comparison to PCA. Both CA and PCA can inhibit mammalian cell growth at concentrations > 600 μM [55]. Based on this data, CA is likely to be a better option for wound healing in Crohn’s fistulas than PCA or Ca-A. Future studies will assess more complex cell/material interactions with these hydrogels and include characterization of additional cell types. However, these preliminary cell studies indicate that the tested hydrogels are cytocompatible candidates for use in biomedical applications, such as Crohn’s fistula filling. 

The PA release rate from the hydrogels also affected antimicrobial properties. All chemically incorporated PAs showed minimal effects on *E. coli* CFUs, whereas physical incorporation of PAs decreased *E. coli* CFU formation. Interestingly, as the number of substituted hydroxyls increases, there is a decrease in overall antimicrobial ability, which corroborates our previous results that showed moderate decreases in antimicrobial activity with increased PA ring pendant groups [16]. In general, PAs represent an alternative option to potentially cytotoxic silver [56,57]. PAs also show effectiveness against drug-resistant strains of *S. aureus* and *S. epidermidis*, thereby proving a potential alternative to traditional antibiotics [16]. The antimicrobial mechanisms of PAs are twofold: (1) PAs can increase the permeability of bacterial outer and plasma membranes, and (2) PAs can bind to bacterial DNA and inhibit cellular functions, both of which can kill the affected cell [37,58,59].

Owing to the poor cytocompatibility and antimicrobial properties of Ca-A, only CA and PCA were examined for biofilm growth. Chemical incorporation did not reduce *S. aureus* biofilm formation. Both CA and PCA reduced biofilm formation at all PA concentrations in physically incorporated hydrogels. This result indicates that the antimicrobial properties of chemically immobilized PAs are reduced as compared with non-immobilized PAs. Future work could examine PA chemical modifications with varied release mechanisms (e.g., Schiff’s base or other reversible click chemistries) [60,61]. These chemistries could be used to limit the burst release of PAs and the negative effects seen here on cell growth. Another option could be to modify PVA chains and incorporate them physically into a crosslinked PVA hydrogel to form a semi-interpenetrating network. By using higher molecular weight chemically unbound polymeric materials, the hydrogels could exhibit more sustained PA release and fewer effects on cellular processes.

All the tested materials show water-responsive shape memory behavior. This property is potentially useful for implantation processes in Crohn’s fistulas. Previous work showed that, by engineering salt-leached foams, these hydrogels can be compressed and stored into a low-profile shape [20]. Ideally, these compressed gels could be delivered to the fistula using a catheter, where they would then expand to recover their primary shape upon exposure to water in the fistula tract and heating to body temperature. This expansion process could fill the fistula tract to enable healing. Here, we show full recovery (unfolding) of non-porous films within 30 min, which can be improved by converting these materials to the porous hydrogels used in our previous work [20]. Future studies will characterize shape change kinetics with sample architectures and geometries that are designed for use in improving implantation processes.

Although we designed these materials for potential use in Crohn’s fistulas, this system could be employed in a range of wound healing applications where infection is of particular concern. For example, ~10,000 people die each year from burn-related infections in the United States alone [62], and biofilms are associated with 60% of burn injury deaths [63]. Additionally, of the many factors that delay healing in chronic wounds, infection plays a significant role and is cited as the likeliest single cause of delayed healing [64]. Furthermore, ~60% of chronic wounds contain biofilm (vs. 6% of acute wounds), which is less amenable to standard antimicrobial treatment [65]. Treating wound infections can involve painful debridement and the use of local antimicrobials, such as iodine or silver, that could hinder healing [66,67,68]. Other internal infections that could be addressed with these antimicrobial hydrogels include bacterial vaginosis, which occurs in ~30% of the female population [69]. Bacterial vaginosis is associated with serious morbidities, such as pain; increased susceptibility to sexually transmitted diseases (e.g., human immunodeficiency virus (HIV)) [70], pregnancy complications [71], and postpartum endometriosis [72]. Thus, these new antimicrobial hydrogel materials could expand antimicrobial dressing options for a range of external and internal wounds in addition to Crohn’s fistula fillers. 

## 5. Conclusions

This study builds upon previous work on shape memory PVA-based hydrogels for Crohn’s fistula healing. With the addition of PAs into a PVA-based hydrogel, we can achieve antimicrobial properties, which may aid fistula healing. Strong antimicrobial and antibiofilm properties were achieved by physically incorporating CA and PCA, and physical incorporation enabled tuning of swelling and mechanical properties. Chemical incorporation did not have a large impact on antimicrobial properties, but it provides a new tool for tuning material properties, including swelling, modulus, and elongation at break. Although the intended application of this material is fistula healing, these strategies could be employed in a range of potential applications, such as biomaterial coatings, chronic wound infections, and vaginal infections. Furthermore, these strategies could be translated to any biomaterial system with hydrogen bonding capabilities, such as poly(ethylene glycol) or polyurethanes. Future work will focus on combining PA incorporation and previously developed biodegradable PVA/cornstarch hydrogels to provide an antimicrobial fistula treatment that degrades during healing.

## Figures and Tables

**Table 1:** Gel fraction (%) and swelling ratio of chemically incorporated PA-PVA hydrogels. PA absorption in physically incorporated PA-PVA hydrogels. *n* = 3; mean ± standard deviation displayed.

	CA			PCA			Ca-A			Control
Amount of PA	1%	2%	5%	1%	2%	5%	1%	2%	5%	0%
Gel Fraction (%) (Chemically Incorporated)	98 ± 2	94 ± 9	100 ± 3	92 ± 18	94 ± 2	83 ± 1	85 ± 2	89 ± 10	77 ± 4	99 ± 4
Swelling Ratio (Chemically Incorporated)	1.46 ± 0.02	2.42 ± 0.67	1.85 ± 0.01	2.14 ± 0.11	2.05 ± 0.01	1.40 ± 0.11	1.85 ± 0.02	1.86 ± 0.68	2.31 ± 0.01	2.87 ± 0.03
PA Absorption (*w*/*w* %) (Physically Incorporated)	25 ± 14	32 ± 1	47 ± 14	37 ± 16	70 ± 17	144 ± 26	35 ± 16	52 ± 27	125 ± 3	N/A

## Data Availability

The data presented in this study are available on request from the corresponding author.
